# Delayed Hemolytic Transfusion Reaction in a Sickle Cell Disease Patient: A Case Report

**DOI:** 10.7759/cureus.12167

**Published:** 2020-12-19

**Authors:** Mohammed Saleh, Vishnu Priya Mallipeddi, Ahmed Ali

**Affiliations:** 1 Internal Medicine, University of Missouri, Columbia, USA; 2 Internal Medicine, Howard University Hospital, Washington, D.C., USA; 3 Oncology, Howard University Hospital, Washington, D.C., USA

**Keywords:** dhtr, sickle cell disease, blood transfusion compl, transfusion reaction

## Abstract

Alloimmunization has been reported in patients with sickle cell disease (SCD). Delayed hemolytic transfusion reaction (DHTR) is one of the complications of alloimmunization. DHTR is of particular clinical significance in this patient population as it may pose a diagnostic and management challenge to most healthcare providers. Symptoms of DHTR are often misinterpreted as pain crisis or worsening of baseline anemia. Furthermore, DHTR may take a turn for the worse in patients with SCD, thereby leading to worsening anemia and hyper-hemolytic crisis.

In this report, we discuss the case of a 33-year-old African female, with hemoglobin SS (Hb SS) SCD and a history of multiple blood transfusions in her home country of Nigeria, who presented to the emergency department with generalized body pain, which was typical of her prior vaso-occlusive crisis (VOC).

The trigger of her crisis was an acute onset of sepsis secondary to *Escherichia coli (E. coli)-*associated pyelonephritis. Owing to a worsening of her VOC and a significant drop in her steady-state Hb levels, she required a blood transfusion of one unit of packed red blood cells (PRBC), which was later complicated by a delayed hemolytic reaction and worsening anemia.

## Introduction

Simple blood transfusion is often regarded as the cornerstone of supportive care in patients with acute sickle cell crisis [1]. Together with other appropriate supportive management measures, blood transfusion can hasten recovery and bring resolution to the crisis state. However, its role in the management of an ongoing delayed hemolytic transfusion reaction (DHTR) is rather controversial [1]. In a certain subset of patients, where the resulting anemia is very severe or is accompanied by a life-threatening complication, blood transfusion becomes the treatment of last resort. In this case of DHTR, this modality of therapy was applied with caution and led to a favorable outcome and resolution of the patient's symptoms.

## Case presentation

A 33-year-old female who had recently emigrated from Nigeria presented to the emergency department with an acute onset of generalized body pain for three days. The pain was more distinctly severe over the right flank and right lower quadrant of the patient’s abdomen. This was associated with an abrupt onset of fever, chills, nausea, and vomiting. In addition, the patient complained of the urinary symptoms of dysuria, frequency, and urgency. Her past medical history was significant for sickle cell anemia with a lifelong history of recurrent vaso-occlusive crisis (VOC) and an acute chest syndrome episode complicated by worsening respiratory failure and requiring admission to the critical care unit. Her most recent blood transfusion had been at the age of 19, as part of the management of her acute chest syndrome episode.

Her general physical exam was significant for hyperpyrexia, tachycardia, and mild bilateral scleral icterus. Her abdomen was moderately tender over the epigastrium, and the right upper and lower quadrants of her abdomen had no guarding or rigidity. Costovertebral angle tenderness was elicited with gentle percussion on the right side of the patient’s back. 

Laboratory studies on admission were significant for leukocytosis, hemoglobin (Hb) level of 6.4 g/dL, and a reticulocyte count of 0.3846 (cells x 10e9/L). Her liver enzymes were slightly elevated with a marked elevation in serum bilirubin and lactate dehydrogenase (LDH). Her urine analysis was positive for numerous bacteria and leukocytes. 

She was subsequently admitted for the management of VOC and sepsis secondary to a presumed urinary tract infection. Intravenous fluid hydration, opioid-based pain control, and empiric levofloxacin therapy were initiated. On day three of admission, her Hb level dropped from 6.4 g/dL on admission to 5.0 g/dL. The patient received one unit of typed and cross-matched packed red blood cells (PRBC). Her blood cultures were positive for extended-spectrum beta-lactamases (ESBL) producing *Escherichia coli (E. coli)*. Levofloxacin was subsequently replaced with meropenem.

Over the course of the ninth and 10th days following her hospitalization, the patient continued to experience worsening of her presenting symptoms. Labs showed an acute drop in Hb to 4.7 g/dL and then to 3.4 g/dL; reticulocytes count was 0.0383 (cells x 10e9/L). LDH level was 1,184 IU/L, with undetectable haptoglobin level; total bilirubin was 5.6 mg/dL, and direct bilirubin was 0.9 mg/dL. Urine studies showed hemoglobinuria. The direct antiglobulin test (DAT) was positive for C3 but not for immunoglobulin G (IgG) (direct antiglobulin studies had been negative on admission). The patient was immediately transferred to the medical intensive care unit (MICU) for the management of DHTR. While in the MICU, she received a 300-mcg dose of darbepoetin subcutaneously and a 55-g dose of intravenous immunoglobulin (IVIg). 

On day 11, and despite the aforementioned interventions, the patient’s Hb continued to drop from 3.4 g/dl to 2.8 g/dl. After a careful assessment of the potential risks and benefits, the decision was made to transfuse one unit of phenotypically matched and crossmatch-compatible PRBC. Following her blood transfusion, the patient showed an adequate clinical response, improvement in her symptoms, rising Hb level, and declining LDH levels. She was discharged on day 18 of her hospitalization with an improved Hb level of 6.7 g/dl.

**Figure 1:**
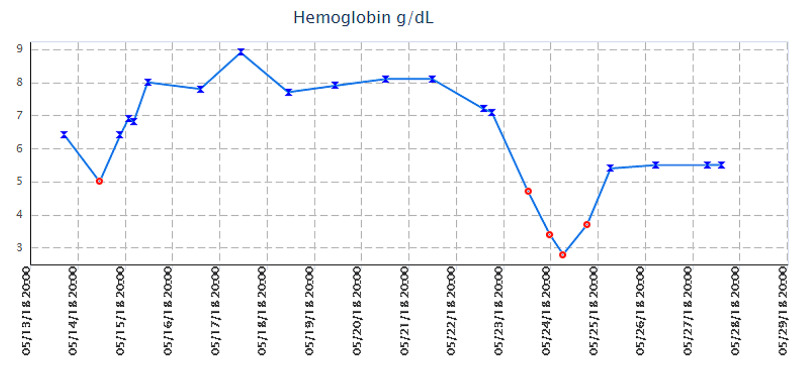
The patient's hemoglobin level trend during the period of hospitalization

She was seen after two weeks of hospital discharge in the hematology clinic. At that time, her Hb was 5.8 g/dl. Reticulocytes count was 0.217 (cells x 10e9/L). Her LDH level was 552 IU/L. Haptoglobin levels were undetectable. Figure 1 shows a graphical representation of her Hb level trend. 

## Discussion

DHTR is a potential sequela of prior blood transfusions. It typically occurs within 3-14 days after receiving a blood transfusion [2] and manifests predominately similar to acute hemolysis (markedly elevated LDH, undetectable haptoglobin, increased total bilirubin, and likely positive DAT) [3].

Prior exposure to blood transfusion causes sensitization to the antigens on RBCs, thereby causing a primary immune response. This process is known as alloimmunization. In this patient, this likely occurred as a complication of a blood transfusion episode when she had been 19 years old. The significant time-lapse had allowed the alloantibodies to drop to undetectable levels, and this may have prevented early detection during conventional screening. Re-exposure to the antigen on donor RBCs led to a secondary immune response, producing IgG antibodies in three to seven days in higher quantities. The donor RBCs that were still present in the recipient’s bloodstream were acutely hemolyzed by the newly formed antibodies [3].

**Figure 2:**
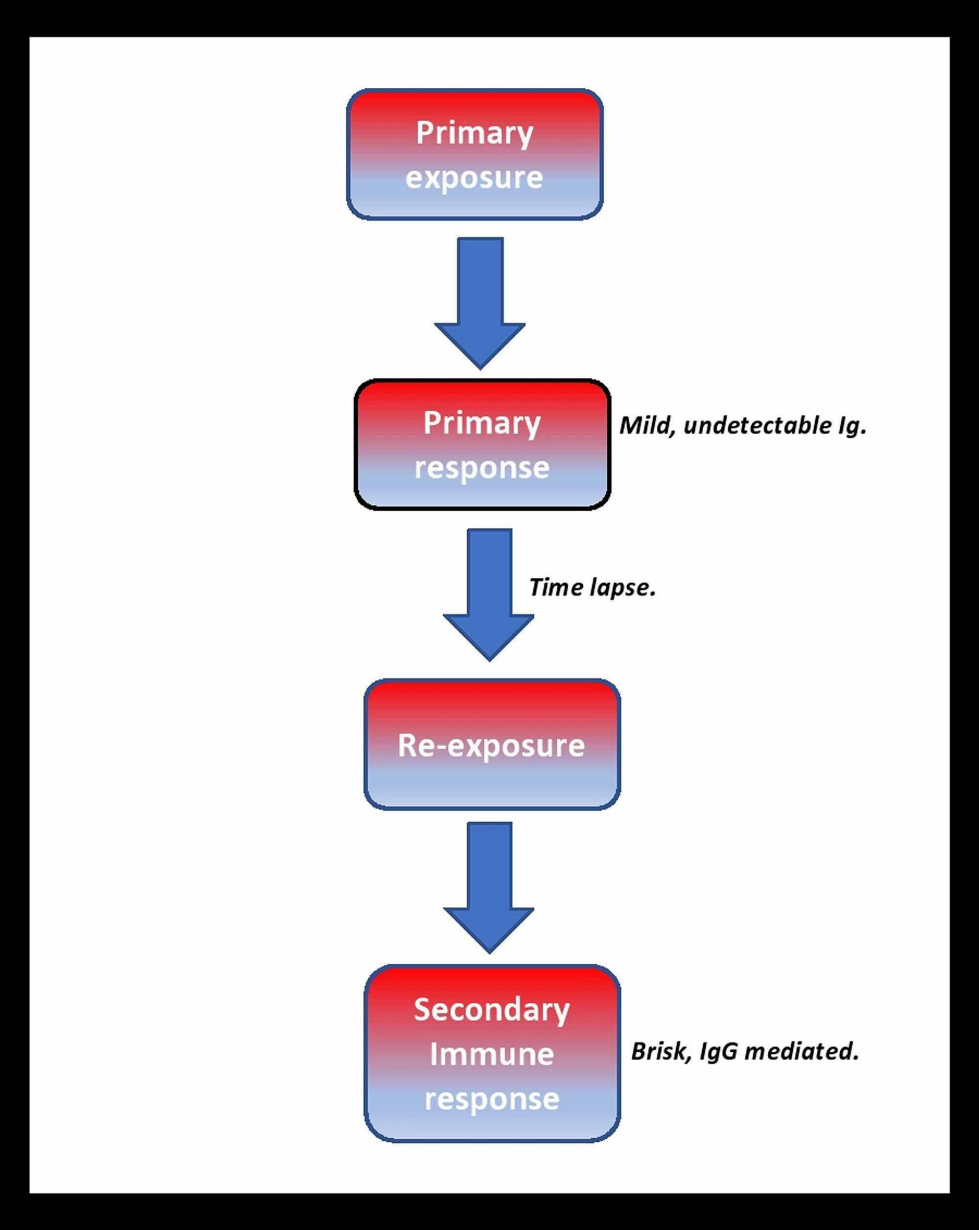
Pathophysiology of delayed hemolytic transfusion reaction Ig: immunoglobulin; IgG: immunoglobulin G

Alloimmunization may also arise due to RBC antigenic variation between Caucasian blood donors and recipients of African descent with sickle cell disease (SCD). This may eventually lead to the production of anti-RBC antibodies by the SCD recipient against the RBC antigens in the donor [3]. Alloimmunization and resultant immune response may cause accelerated destruction of both donor RBCs and recipient RBCs, a phenomenon known as bystander hemolysis [4]. Figure 2 depicts the pathophysiology of DHTR.

The incidence of reported cases of DHTR in patients with SCD is widely believed to be an underestimation of the actual rates of this complication [5]. Cases of DHTR in this population are often missed, as it may produce a similar clinical picture to that of worsening acute VOC. Furthermore, the post-transfusion screening test tends to yield false-negative results [5].

A 30-month single-center prospective study was conducted in 2017, which involved following up on 694 transfusion episodes administered to 301 patients to estimate the incidence of DHTR in adult sickle cell patients. The transfusions administered were further classified into occasional transfusion episodes (OTEs) and those that were part of a chronic transfusion program (CTE) [6]. During the follow-up, 15 cases of DHTR were recorded, exclusively after OTEs. DHTR incidence was 4.2% per OTE (95% CI [2.6; 6.9]) and 6.8% per patient during the study period of 30 months (95% CI [4.2; 11.3]) [6]. Data from previous studies suggest that the incidence of DHTR is anywhere from 4% to 11% [3,7]. This represents only 0.05% of the cases diagnosed with alloimmunization [8]. Patients with suspected DHTR should be evaluated with a direct antigen (Coombs’) test [2]. Once the antigen is identified, it should be documented to be avoided in future transfusions.

The cornerstone of the management of DHTR is immunosuppressive therapy. Many SCD patients with DHTR have been successfully treated with IVIg [3]. Erythropoietin should be administered to all patients with DHTR and reticulocytopenia [9], which is not an uncommon finding in patients with DHTR despite the ongoing RBC hemolysis.

The general consensus is to withhold further transfusions to avoid accelerated hemolysis [9]. However, in certain cases with profound anemia and subsequently worsening hypoxemia or heart failure, the administration of additional transfusions may become unavoidable as a measure of last resort [9]. In our patient, a severe drop in hemoglobin levels to <3 g/dl and impending cardiopulmonary failure were the grounds for our decision to administer a further blood transfusion.

## Conclusions

DHTR is a rare but serious complication that could potentially arise from recurrent blood transfusions in patients with SCD. Diagnosing DHTR can be challenging, especially in the setting of acute VCO. With a careful assessment of risks and benefits, blood transfusion can be used as a treatment of last resort when other conservative methods fail, or when cardiopulmonary compromise becomes evident.
